# Astragaloside IV confers vascular protection after traumatic brain injury through an Nrf2-dependent mechanism involving VWF

**DOI:** 10.1016/j.ibneur.2026.06.017

**Published:** 2026-09-04

**Authors:** Shiyao Feng, Boyu Sun, Mingkang Li, Longlong Tian, Guozhu Sun

**Affiliations:** Department of Neurosurgery, The Second Hospital of Hebei Medical University, Shijiazhuang, China

**Keywords:** Traumatic brain injury, Astragaloside IV, Vascular protection, Nrf2, VWF

## Abstract

Traumatic brain injury (TBI) is a major cause of neurological disability worldwide, yet effective pharmacological therapies remain limited. Astragaloside IV (AS-IV), a major active component derived from Astragalus membranaceus, exhibits neuroprotective properties, but the mechanisms underlying its effects after TBI remain unclear. In this study, we investigated the role of nuclear factor erythroid 2 related factor 2 (Nrf2) signaling in AS-IV mediated neuroprotection using a mouse TBI model. AS-IV significantly improved neurological function and attenuated neuronal injury after TBI, accompanied by enhanced Nrf2 nuclear translocation. The anti-inflammatory and antioxidant effects of AS-IV were partially attenuated in Nrf2-deficient mice, whereas its protective effects on brain edema, blood-brain barrier integrity, and cerebral blood flow were nearly completely abolished. Further analyses identified von Willebrand factor (VWF) as a vascular related gene associated with Nrf2 dependent vascular protection following TBI. These findings indicate that the vascular protective effects of AS-IV after TBI rely on Nrf2 signaling, with VWF potentially contributing to this process.

## Introduction

Traumatic brain injury is one of the most common neurological disorders worldwide and represents a serious public health problem (Maas et al., 2022). TBI is commonly divided into primary injury and secondary injury. Primary injury results from the initial mechanical insult and is largely irreversible, whereas secondary injury develops through a complex cascade of pathophysiological processes such as cerebral edema, blood-brain barrier disruption, excitotoxicity, oxidative stress, and cell death, ultimately leading to neurological dysfunction (Freire et al., 2023). Secondary brain injury is a central focus of current therapeutic strategies for TBI. Although numerous interventions have been developed, effective clinical therapies remain limited.

Astragaloside IV (AS-IV) is a triterpenoid saponin and one of the major active components derived from Astragalus membranaceus, exhibiting multiple pharmacological activities including anti-inflammatory, antioxidant, neuroprotective, anti-fibrotic, and anti-tumor properties (Liang et al., 2023). Previous studies have shown that AS-IV exerts neuroprotective effects in several neurological disorders, such as ischemic stroke, subarachnoid hemorrhage, and neurodegenerative diseases (Yao et al., 2023). More recently, AS-IV has also been investigated in experimental models of TBI. Studies have shown that AS-IV alleviates brain injury and improves neurological function by attenuating microglial activation (Yang et al., 2014). It has also been reported that AS-IV attenuates neuroinflammation and improves neurological outcomes by modulating the PERK-eIF2α-ATF4 signaling pathway (Zhao et al., 2024). However, the molecular mechanisms underlying the neuroprotective effects of AS-IV after TBI remain incompletely understood.

Nuclear factor erythroid 2 related factor 2 (Nrf2) is a conserved Cap ‘n’ Collar basic leucine zipper transcription factor that regulates cellular stress responses and maintains redox homeostasis (Bonay, 2024). Increasing evidence suggests that Nrf2 activation mitigates TBI-induced injury by suppressing oxidative stress, neuroinflammation, and neuronal apoptosis while preserving blood-brain barrier integrity, thereby improving neurological outcomes (Wu et al., 2022). AS-IV has been shown to activate the Nrf2 signaling pathway and promote Nrf2 nuclear translocation (Yu et al., 2021). Correspondingly, AS-IV exerts neuroprotective effects in neurological disorders such as stroke (Zhang et al., 2023). However, studies on whether the protective effects of AS-IV after TBI involve Nrf2 signaling remain limited, and the underlying mechanisms remain unclear.

In the present study, we investigated the role of Nrf2 signaling in AS-IV mediated neuroprotection after TBI. To address this question, we evaluated the neuroprotective effects of AS-IV in both wild type and Nrf2-deficient mice subjected to TBI. Our findings provide novel insights into the molecular mechanisms underlying AS-IV-mediated neuroprotection following TBI.

## Methods

### Animals

The animal procedures for this investigation conformed to the Guide for the Care and Use of Laboratory Animals (National Research Council) and were approved by the Institutional Animal Care and Use Committee of the Second Hospital of Hebei Medical University (Approval No. 2026-AE392). Male ICR mice (8–10 weeks old, 28–32 g) were provided by Beijing Vital River Laboratory Animal Technology Co., Ltd. Age- and weight-matched male Nrf2⁻/⁻ mice on an ICR background were provided by Professor Chunyan Li (Department of Neurology, Second Hospital of Hebei Medical University, Shijiazhuang, China). Only male mice were used to reduce biological variability in mechanistic investigations. The mice were housed under specific pathogen-free (SPF) conditions with free access to water and food, at a temperature of 23°C ± 2°C, humidity of 60%–70%, and a 12-hour light/dark cycle. Mice were acclimatized for 7 days before the experiment.

### Experimental design and animal allocation

The study comprised two independent experiments, and a total of 170 male mice were used. In Experiment 1, mice were randomly assigned to four groups: Sham, TBI, TBI + vehicle, and TBI + AS-IV. Three cohorts were included: Cohort 1 for behavioral assessments (n = 5 per group), Cohort 2 for histological analyses (n = 3 per group), and Cohort 3 for Western blot analyses (n = 3 per group). A total of 44 mice were used in Experiment 1. In Experiment 2, mice were randomly assigned to six groups: Sham, TBI, TBI + AS-IV, Nrf2⁻/⁻, Nrf2⁻/⁻ + TBI, and Nrf2⁻/⁻ + TBI + AS-IV. Five cohorts were included: Cohort 1 for laser speckle imaging, ELISA, and oxidative stress assays (n = 5 per group); Cohort 2 for brain water content measurement (n = 5 per group); Cohort 3 for Evans Blue extravasation (n = 5 per group); Cohort 4 for qPCR and Western blot analyses (n = 3 per group); and Cohort 5 for immunofluorescence staining (n = 3 per group). A total of 126 mice were used in Experiment 2, including 63 wild-type and 63 Nrf2⁻/⁻ mice.

### Genotyping of Nrf2⁻/⁻ mice

Genomic DNA was extracted from mouse tail tips using the NaOH based method (Lopez, 2012). PCR amplification was performed using 2 ×Taq Master Mix (P222–01, Vazyme, China) on a thermal cycler (Mastercycler nexus GSX1, Eppendorf, Germany). The primer sequences used for genotyping were as follows: NRF5 (in Nrf2 gene), 5’-TGGACGGGACTATTGAAGGCTG−3’; NAS (in Nrf2 gene), 5’-GCCGCCTTTTCAGTAGATGGAGG−3’; and NLACZ (in LacZ gene), 5’-GCGGATTGACCGTAATGGGATAGG−3’. PCR products were separated on 1.5% agarose gels and visualized using a gel documentation system (GBOX-F3, Gene Company Limited, China). Genotypes were determined based on band patterns. All genotyping procedures were performed by investigators blinded to group allocation.

### TBI model

The mouse TBI model was established using a weight drop apparatus. Mice were anesthetized with isoflurane (No. G45993, EZVET) and secured in a stereotaxic frame. A circular craniotomy measuring 3 mm in diameter was performed over the left parietal bone, centered 4.0 mm posterior to bregma and 4.0 mm lateral to the midline, with the dura kept intact. A 40 g weight was dropped from a height of 20 cm onto the exposed cortical surface to induce injury. The scalp was sutured immediately after impact. The criteria for successful model establishment were a visible cortical contusion and an mNSS score ≥ 8 at 30 min post injury. Sham operated mice underwent identical surgical procedures without weight impact.

### Drug administration

Astragaloside IV (AS-IV) (Cayman, 12069, purity >95%), with a molecular weight of 784.97 and CAS # 84687–43–4, was solubilized according to the instructions. The drug was dissolved in DMSO and further diluted with PBS (pH 7.2) to a final concentration of 0.5 mg/mL. AS-IV was administered by intraperitoneal injection at a dose of 20 mg/kg (Yang et al., 2014) at 1 h after TBI and subsequently once daily on post injury day 1 and day 2. Vehicle treated mice received intraperitoneal injections of the solvent with an identical composition at the same time points.

### Brain tissue preparation

For molecular analysis, mice were euthanized and the brains were rapidly removed. Pericontusional cortical tissues (approximately 2–3 mm surrounding the contusion core) at the level of the impact center were carefully dissected. The samples were immediately frozen and stored at −80 °C until use. These tissues were used for Western blotting, quantitative PCR, ELISA, and oxidative stress assays.

For histological analysis, mice were perfused with phosphate buffered saline followed by 4% paraformaldehyde. Whole brains were removed and further fixed in 4% paraformaldehyde overnight. Paraffin embedded tissues were prepared for Nissl staining, and cryo embedded tissues were prepared for immunofluorescence and TUNEL staining. Coronal sections were obtained at the level of the impact center. Regions of interest (ROIs) for quantitative analysis were defined as the ipsilateral pericontusional cortex (approximately 2–3 mm surrounding the contusion core).

### Neurological evaluation

Neurological evaluation was assessed using the modified Neurological Severity Score (mNSS) (Liu et al., 2023). The mNSS is an 18 points composite scale evaluating motor, sensory, balance, and reflex functions, with 1 point assigned for each abnormal response. Higher scores indicate more severe neurological deficits. All assessments were performed by two independent investigators blinded to group allocation.

### Rotarod test

The rotarod test was used to assess motor coordination and balance in mice. Mice were trained for five consecutive days before TBI. During training, the rotation speed was maintained at 4 RPM. Only mice able to remain on the rod for more than 60 s were included in the study. For testing, mice were placed on the rotating rod, and the speed was gradually increased from 4 RPM to 40 RPM over 4 min. The latency to fall was recorded. The trial ended when the mouse fell or when the 4 min period elapsed (Fan et al., 2022). The average latency from three trials was calculated for each mouse. All assessments were performed by two independent investigators blinded to group allocation.

### Grip strength test

Grip strength was measured using a digital grip strength meter (GS3, BIOSEB, Vitrolles, France). Mice were habituated to the apparatus for five days before testing. During the test, each mouse grasped the metal grid with its forepaws and was gently pulled backward along the body axis until release. Grip strength was recorded in grams (McBean et al., 2021). Each mouse performed three trials at intervals of at least 15 min, and the mean value was calculated. All assessments were performed by two independent investigators blinded to group allocation.

### Terminal deoxynucleotidyl transferase dUTP nick-end labeling (TUNEL) assay

Frozen brain sections (10 μm) were stained using a TUNEL assay kit (FITC-labeled, KeyGEN, KGA1406). Nuclei were counterstained with DAPI. Fluorescent images were captured using a fluorescence microscope (Axiovert A1, Zeiss AG, Germany). The apoptosis index was calculated as the ratio of TUNEL positive cells to total DAPI positive cells using Fiji software. All assessments were performed by two independent investigators blinded to group allocation.

### Nissl staining

Paraffin embedded brain sections (4 μm) were stained using a Nissl staining kit (Solarbio, G1436) according to the manufacturer’s instructions. Images were captured using a light microscope (Axiovert A1, Zeiss AG, Germany). The average optical density (AOD) of Nissl staining was measured using Fiji software. All assessments were performed by two independent investigators blinded to group allocation.

### Western blot analysis

Pericontusional cortical tissues were lysed in RIPA buffer supplemented with protease inhibitors to obtain total protein extracts. Nuclear and cytoplasmic proteins were isolated using a nuclear protein extraction kit (Applygen, P1202) according to the manufacturer’s instructions. Protein concentrations were determined using a BCA assay. Equal amounts of protein were denatured, separated by SDS-PAGE, and transferred onto PVDF membranes. After blocking, membranes were incubated overnight at 4 ℃ with primary antibodies, followed by appropriate secondary antibodies. Protein signals were detected using an Odyssey infrared imaging system (LI-COR Biosciences, USA). Band intensities were quantified using Fiji software and normalized to β-actin or Histone H3 as appropriate. Primary antibodies were as follows: Nrf2 (1:1000, MBL, M200–3); HO−1 (1:1000, Proteintech, 10701–1-AP); NQO1 (1:1000, Proteintech, 11451–1-AP); VWF (1:1000, Proteintech, 83854–2-RR); ZO−1 (1:1000, Proteintech, 21773–1-AP); β-actin (1:3000, Proteintech, 20536–1-AP); and Histone H3 (1:1000, Proteintech, 17168–1-AP). Secondary antibodies were infrared fluorophore conjugated antibodies (1:10000, Affinity Biosciences, S0001 and S0002).

### ELISA analysis

Samples were homogenized and the supernatants were collected for analysis. The concentrations of TNF-α, IL−1β, IL−6, and IL−10 were determined using ELISA kits (ABclonal, RK00027, RK00006, RK00008, and RK00016) according to the manufacturer’s instructions. Absorbance values were measured using a multimode microplate reader (TECAN SPARK 10 M). Cytokine levels were normalized to total protein content.

### Measurement of oxidative stress related indicators

Samples were homogenized and the supernatants were collected for analysis. The activities or levels of CAT, SOD, GSH, and MDA were determined using commercial assay kits (Boxbio, AKAO003–1M, AKAO001M, AKPR008M, and AKFA013M). Absorbance values were measured using a multimode microplate reader (TECAN SPARK 10 M). Results were normalized to total protein content.

### Brain water content

Brain water content was determined using the wet-dry weight method. The ipsilateral hemisphere was rapidly dissected and immediately weighed to obtain the wet weight. Samples were then dried at 100 °C for 24 h, and the dry weight was recorded. Brain water content was calculated using the following formula: [(wet weight - dry weight)/wet weight] × 100%. All assessments were performed by two independent investigators blinded to group allocation.

### Evans blue extravasation assay

Blood-brain barrier permeability was assessed using 0.5% Evans Blue dye (Solarbio, G1810). Mice were injected intravenously at a dose of 2 mL/kg. One hour after injection, mice were sacrificed and pericontusional cortical tissues (approximately 2–3 mm surrounding the contusion core) were collected and weighed. Tissues were homogenized in 50% trichloroacetic acid and centrifuged, and the supernatant was collected. Absorbance was measured at 620 nm using a microplate reader. Evans Blue concentration was calculated from a standard curve and expressed as μg/g tissue.

### Laser speckle contrast imaging

Cortical blood flow was measured using a laser speckle blood flow monitoring system (PeriCam PSIHR, PSIH−10129) with PIMSoft software (Version I·S). Under anesthesia, the scalp was incised along the midline to expose the skull. Mice were fixed in a stereotaxic frame, and cerebral blood flow over the cortical surface was recorded. Imaging parameters were kept constant throughout the experiment. Relative perfusion values were analyzed using PIMSoft software, and the region of interest was defined as the impact region.

### RNA sequencing and bioinformatics analysis

RNA sequencing of pericontusional cortical tissues from WT + TBI and Nrf2⁻/⁻ + TBI mice was performed by Biomarker Biotechnology Co., Ltd. Differential expression analysis was conducted using the DESeq2 package in R version 4.4.2. Genes with an adjusted P value < 0.05 and an absolute log2 fold change ≥ 1 were considered differentially expressed. Volcano plots and heatmaps of the top 20 differentially expressed genes were generated in R. Gene set enrichment analysis (GSEA) was performed using the Hallmark gene sets (mh.all.v2025.1.Mm.symbols.gmt). All sequencing datasets generated in this study have been deposited in the NCBI Sequence Read Archive under BioProject accession number PRJNA1337780.

### Real-time quantitative PCR

Total RNA was extracted using an RNA extraction kit (Zhongshi, ZS-M11005) and reverse transcribed into complementary DNA with a cDNA synthesis kit (Zhongshi, ZS-M14003). Quantitative PCR was conducted using SYBR Green premix (Zhongshi, ZS-M13002) on a LightCycler 480 II system (Roche Diagnostics). Relative mRNA expression levels were calculated using the 2^(-ΔΔCt) method. Primer sequences (5’ to 3’) were as follows: VWF (F AACAGACGATGGTGGACTCAGC, R CGATGGACTCACAGGAGCAAGT); CD248 (F TTGATGGCACCTGGACAGAGGA, R TCCAGGTGCAATCTCTGAGGCT); Timp1 (F TCTTGGTTCCCTGGCGTACTCT, R GTGAGTGTCACTCTCCAGTTTGC); Tagln (F GCAGATGGAACAGGTGGCTCAA, R CCCAAAGCCATTAGAGTCCTCTG); and GAPDH (F CATCACTGCCACCCAGAAGACTG, R ATGCCAGTGAGCTTCCCGTTCAG).

### Immunofluorescence staining

Frozen brain sections (10 μm) were permeabilized with 0.5% Triton X−100 and blocked with 10% goat serum, followed by incubation with primary antibodies overnight at 4 ℃ and fluorophore conjugated secondary antibodies at room temperature in the dark. Nuclei were counterstained with DAPI. Images were captured using a fluorescence microscope (Axiovert A1, Zeiss AG, Germany). The mean fluorescence intensity was measured using Fiji software. All assessments were performed by two independent investigators blinded to group allocation. Primary antibodies were as follows: VWF (1:200, Proteintech, 83854–2-RR); ZO−1 (1:200, Proteintech, 21773–1-AP); and CD31 (1:400, Proteintech, 66065–2-Ig). Secondary antibodies were Dylight 488 goat anti-rabbit IgG (1:200, Abbkine, A23220) and Dylight 594 goat anti-mouse IgG (1:200, Abbkine, A23410).

### Statistical analysis

Sample size (n) represents the number of independent animals used in each experiment. Statistical analyses were performed using GraphPad Prism (v10.4.1). Data are presented as mean ± SD. Comparisons among multiple groups were analyzed using one way analysis of variance followed by Tukey multiple comparisons test. In some experiments, data were normalized to the sham or control group. A P value < 0.05 was considered statistically significant.

## Results

### AS-IV ameliorates neurological dysfunction after TBI

**Fig. 1:**
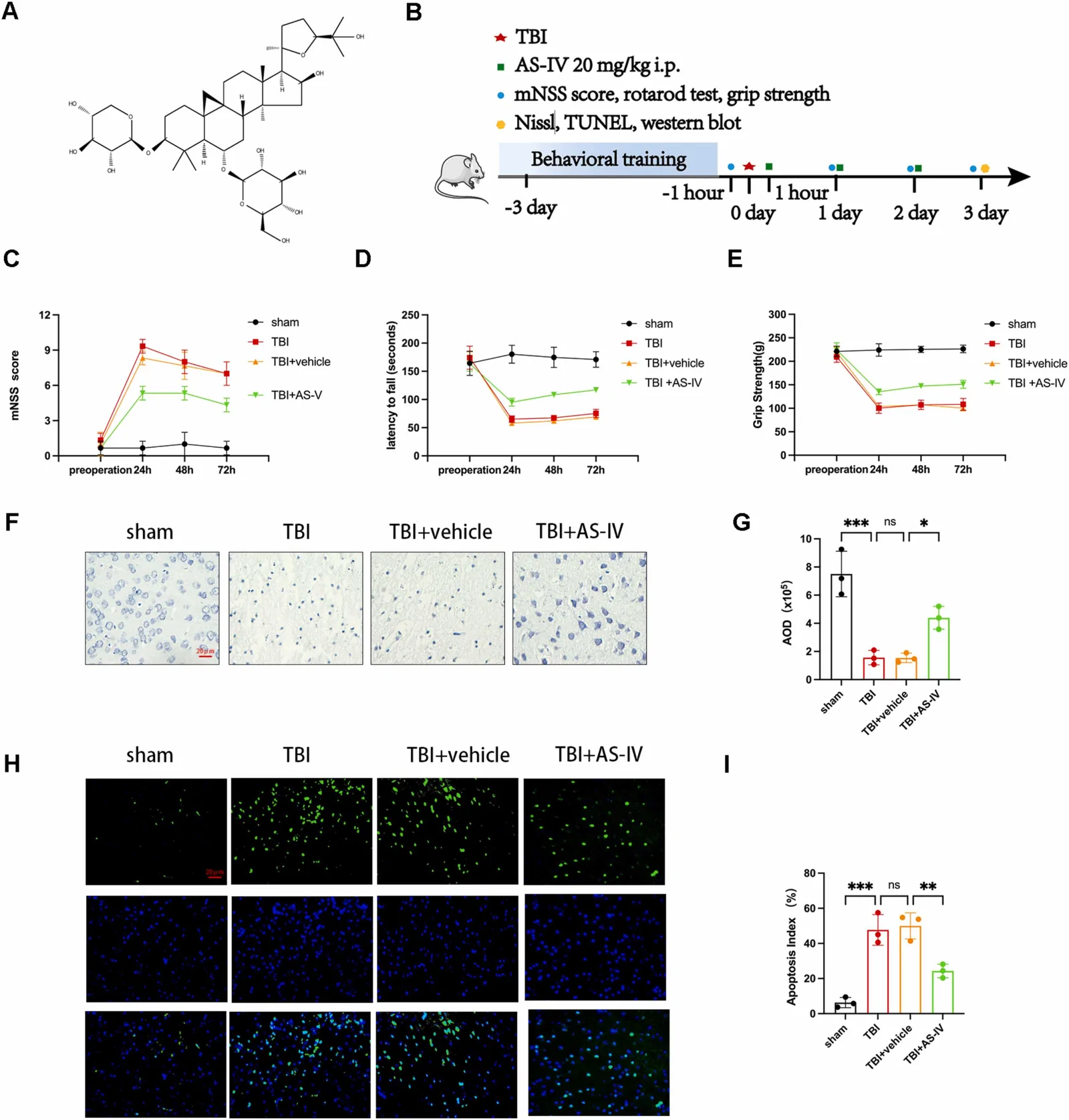
Behavioral and histological alterations in TBI mice treated with AS-IV. (A) Chemical structure of AS-IV. (B) Experimental timeline showing TBI induction, AS-IV administration, behavioral assessments, and tissue collection at the indicated time points. (C–E) Behavioral assessments, including (C) mNSS score, (D) rotarod test, and (E) grip strength test, performed preoperatively and at 24 h, 48 h, and 72 h after TBI (n = 5 per group). (F–G) Representative Nissl staining images and quantitative analysis of neuronal morphology in the pericontusional cortex at 3 days post-TBI (n = 3 per group). Scale bar, 20 μm. (H–I) Representative TUNEL staining images and quantitative analysis of the percentage of TUNEL-positive cells in the pericontusional cortex at 3 days post-TBI (n = 3 per group). Scale bar, 20 μm. Data are presented as mean ± SD. *p < 0.05, **p < 0.01, ***p < 0.001; ns, not significant.

The chemical structure of AS-IV and the experimental timeline are presented in Fig. 1A–B. Mice received three intraperitoneal injections of AS-IV at 1 h after TBI and on post-injury days 1 and 2. Neurological function was assessed on days 1, 2, and 3 after injury, and histological analyses were conducted on day 3. TBI resulted in marked neurological impairment, as evidenced by significantly elevated mNSS scores at all examined time points. AS-IV treatment significantly reduced mNSS scores compared with the TBI group (Fig. 1C). Consistently, TBI impaired motor coordination and balance, reflected by reduced rotarod performance, whereas AS-IV administration significantly improved motor performance (Fig. 1D). Forelimb grip strength was also decreased after injury and was partially restored by AS-IV treatment (Fig. 1E). Nissl staining revealed pronounced neuronal loss and structural disruption in the pericontusional cortex after TBI. AS-IV treatment markedly alleviated neuronal damage (Fig. 1F–G). TBI markedly increased the number of TUNEL-positive cells in the pericontusional cortex, and this increase was significantly attenuated by AS-IV (Fig. 1H–I). Together, these findings demonstrate that AS-IV improves neurological function and mitigates neuronal injury following TBI.

### AS-IV augments TBI-induced Nrf2 nuclear translocation

**Fig. 2:**
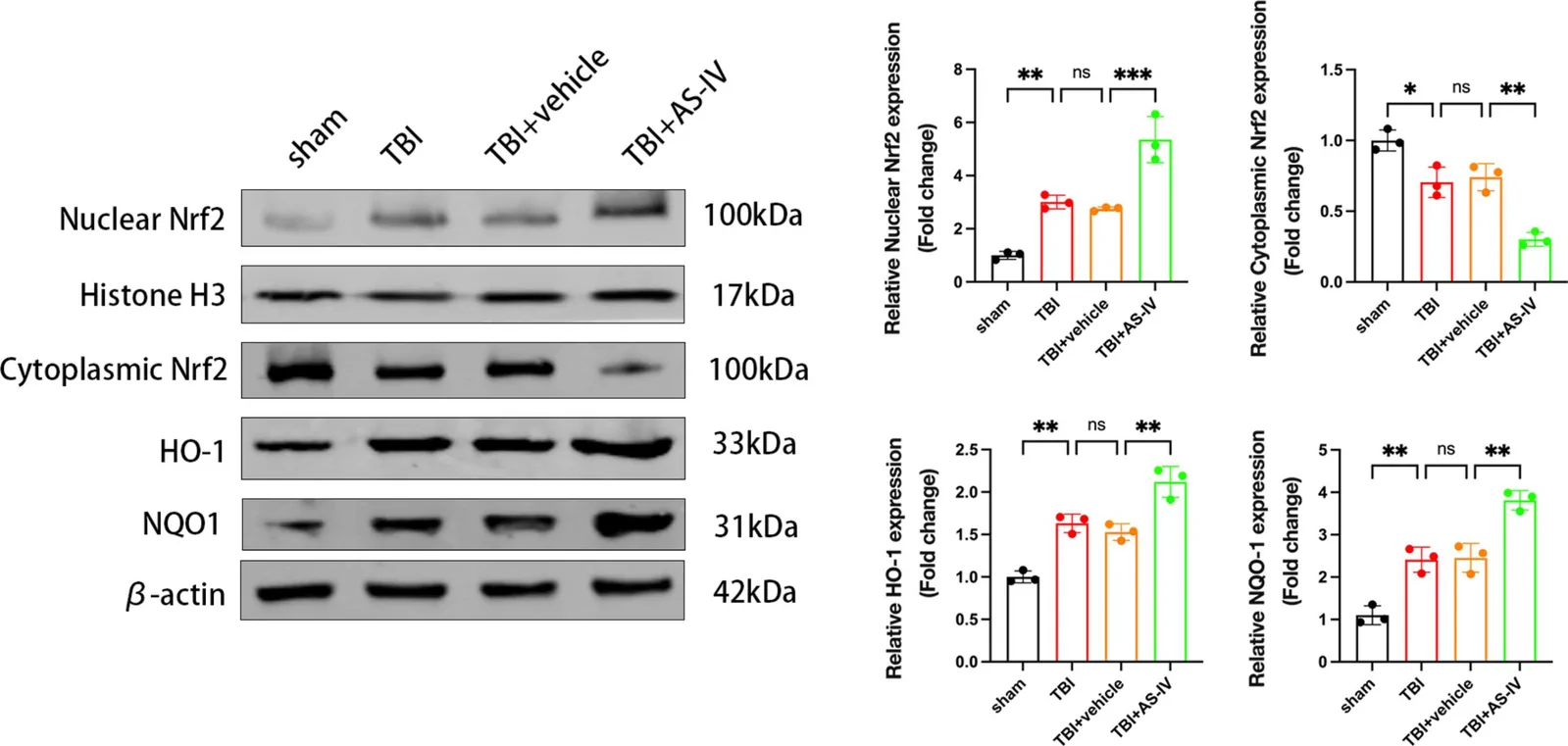
Nrf2 nuclear translocation and downstream antioxidant protein expression after TBI and AS-IV treatment. Western blot and quantitative analysis of nuclear Nrf2, cytoplasmic Nrf2, HO−1, and NQO1 expression in the pericontusional cortex at 3 days post-TBI. Nuclear Nrf2 levels were normalized to Histone H3, and cytoplasmic Nrf2, HO−1, and NQO1 levels were normalized to β-actin (n = 3 per group). Data are presented as mean ± SD. *p < 0.05, **p < 0.01, ***p < 0.001; ns, not significant.

Nuclear and cytoplasmic proteins were extracted from the pericontusional cortex on day 3 after TBI. Western blot analysis showed that TBI increased nuclear Nrf2 expression while reducing cytoplasmic Nrf2 levels. AS-IV further promoted Nrf2 translocation to the nucleus. The downstream antioxidant proteins HO−1 and NQO1 were elevated after TBI and were further increased following AS-IV administration (Fig. 2).

### Nrf2 deficiency abrogates the vascular protective effects of AS-IV after TBI

**Fig. 3:**
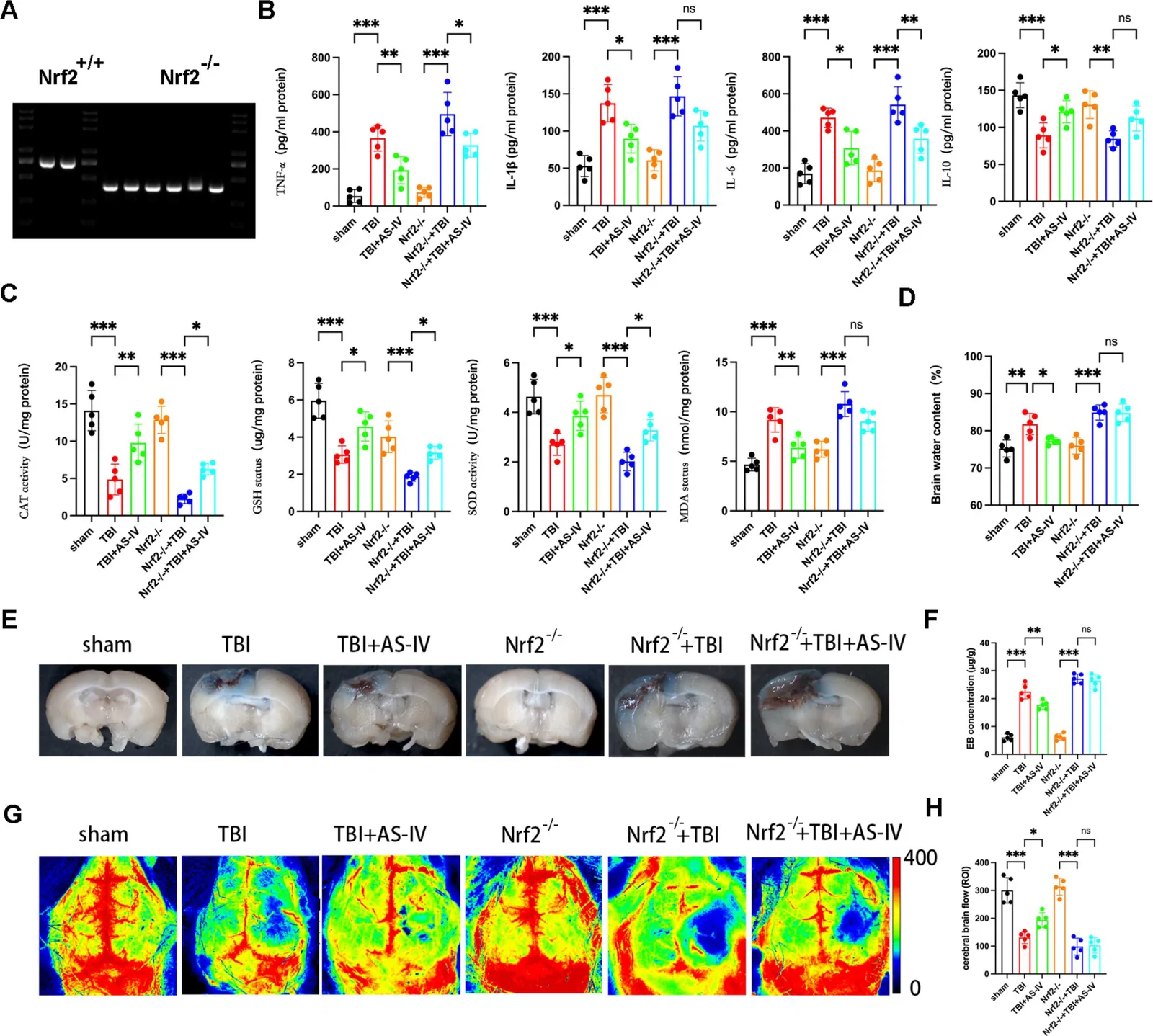
Effects of AS-IV on inflammatory responses, oxidative stress, and vascular parameters in WT and Nrf2⁻/⁻ mice after TBI. (A) PCR genotyping of WT and Nrf2⁻/⁻ mice showing a 700 bp band in WT mice and a 400 bp band in Nrf2⁻/⁻ mice. (B) Levels of TNF-α, IL−1β, IL−6, and IL−10 in the pericontusional cortex (n = 5 per group). (C) CAT, GSH, SOD, and MDA levels in the pericontusional cortex (n = 5 per group). (D) Brain edema assessed by brain water content at 3 days post-TBI (n = 5 per group). (E–F) Representative whole brain images and quantitative analysis of Evans Blue concentration (n = 5 per group). (G–H) Representative cerebral blood flow maps and quantitative analysis of cerebral blood flow within the ROI (n = 5 per group). Data are presented as mean ± SD. *p < 0.05, **p < 0.01, ***p < 0.001; ns, not significant.

Nrf2⁻/⁻ mice were used to determine the role of Nrf2 in AS-IV–mediated protection after TBI. Genotyping verified the knockout model, showing a 700 bp band in WT mice and a 400 bp band in Nrf2⁻/⁻ mice (Fig. 3A). WT and Nrf2⁻/⁻ mice were assigned to sham, TBI, and TBI + AS-IV groups. TBI markedly enhanced the inflammatory response in the pericontusional cortex, as reflected by increased TNF-α, IL−1β, and IL−6 levels and reduced IL−10. AS-IV treatment significantly reversed these alterations in WT mice. In Nrf2⁻/⁻ mice, AS-IV partially reversed these changes, although the effects were less pronounced than in WT mice (Fig. 3B). Oxidative stress was similarly aggravated after TBI, as indicated by decreased SOD, CAT, and GSH levels and increased MDA. AS-IV significantly ameliorated these alterations in WT mice. Its antioxidant effects were attenuated in Nrf2⁻/⁻ mice (Fig. 3C). TBI resulted in brain edema, increased Evans Blue extravasation, and reduced cerebral blood flow. AS-IV markedly improved these vascular impairments in WT mice. In contrast, AS-IV failed to improve brain edema, blood-brain barrier permeability, or cerebral perfusion in Nrf2⁻/⁻ mice (Fig. 3D–H).

### VWF is identified as a candidate mediator associated with the loss of AS-IV-induced vascular protection in Nrf2-deficient mice

**Fig. 4:**
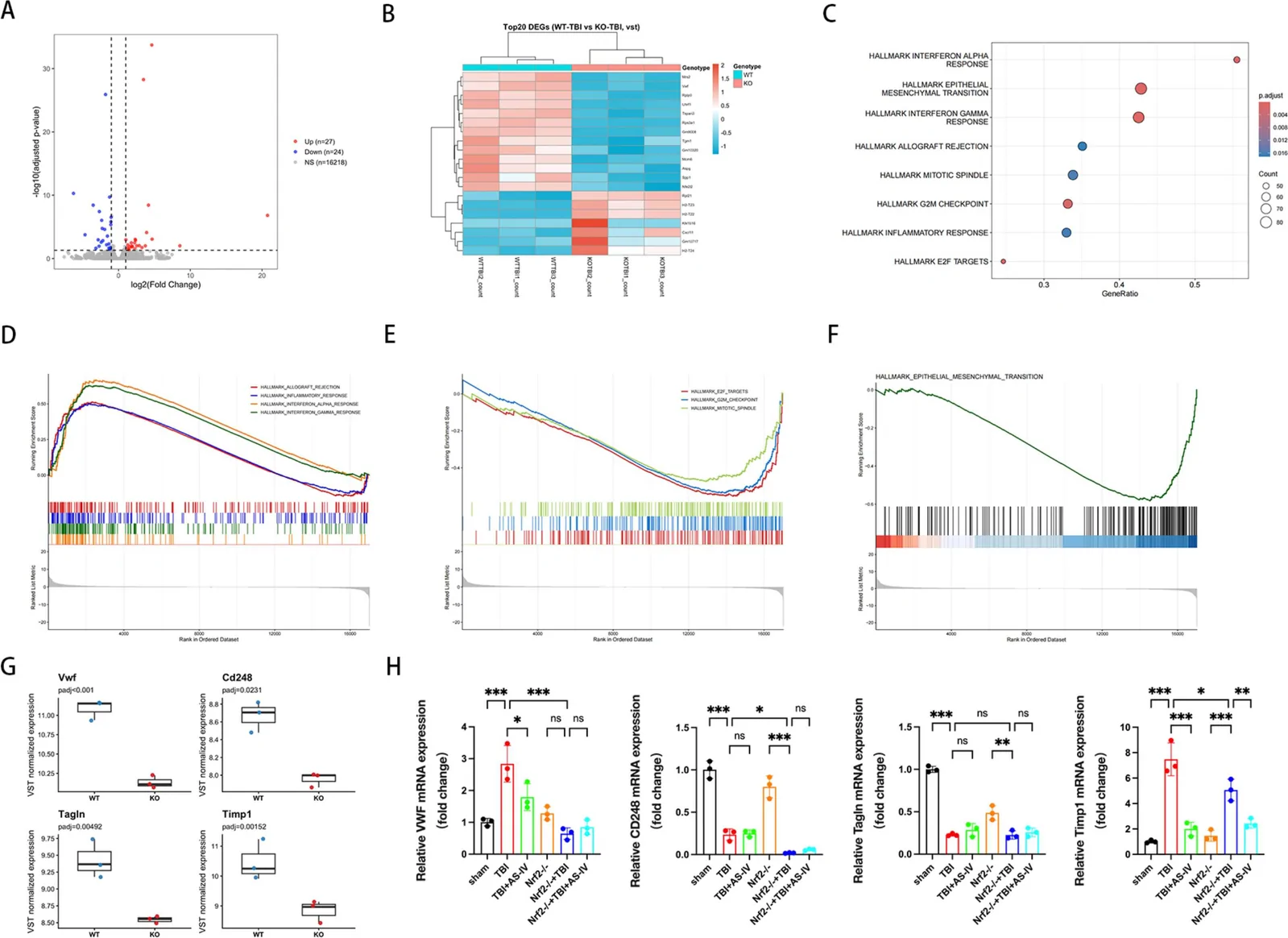
VWF is identified as a candidate mediator associated with the loss of AS-IV-induced vascular protection in Nrf2-deficient mice. (A) Volcano plot of differentially expressed genes (DEGs) between WT + TBI and Nrf2⁻/⁻ + TBI mice. (B) Heatmap of the top 20 DEGs. (C) GSEA dot plot of enriched pathways. (D–F) Representative GSEA enrichment curves for inflammatory and immune response pathways (D), cell cycle pathways (E), and epithelial mesenchymal transition (F). (G) Box plots of Vwf, Cd248, Timp1, and Tagln expression between WT + TBI and Nrf2⁻/⁻ + TBI mice. (H) qPCR analysis of Vwf, Cd248, Tagln, and Timp1 mRNA levels (n = 3 per group). Data are presented as mean ± SD. *p < 0.05, **p < 0.01, ***p < 0.001; ns, not significant.

RNA sequencing identified 51 differentially expressed genes between WT + TBI and Nrf2⁻/⁻ + TBI mice, including 27 upregulated and 24 downregulated genes in Nrf2⁻/⁻ mice (Fig. 4A). A heatmap of the top 20 DEGs further illustrated clear transcriptional differences between the two groups (Fig. 4B). GSEA revealed significant enrichment in inflammatory and immune responses, cell cycle regulation, and epithelial mesenchymal transition (Fig. 4C). Epithelial-mesenchymal transition has been closely associated with vascular remodeling and endothelial dysfunction. Representative enrichment curves for these categories are shown in Fig. 4D–F.

Among the DEGs, four vascular related genes were identified: Vwf, Cd248, Timp1, and Tagln, as shown in the box plots (Fig. 4G). qPCR analysis confirmed these expression changes and further evaluated their responsiveness to AS-IV across genotypes (Fig. 4H). In WT mice, Vwf expression was markedly increased after TBI and significantly reduced by AS-IV treatment, whereas this regulatory effect was absent in Nrf2⁻/⁻ mice. Although Cd248 and Tagln expression was altered following TBI, neither gene was significantly modulated by AS-IV treatment in WT mice. Timp1 was responsive to AS-IV in WT mice, with similar regulation observed in Nrf2⁻/⁻ mice.

### Nrf2 deficiency abrogates AS-IV mediated regulation of VWF expression after TBI

**Fig. 5:**
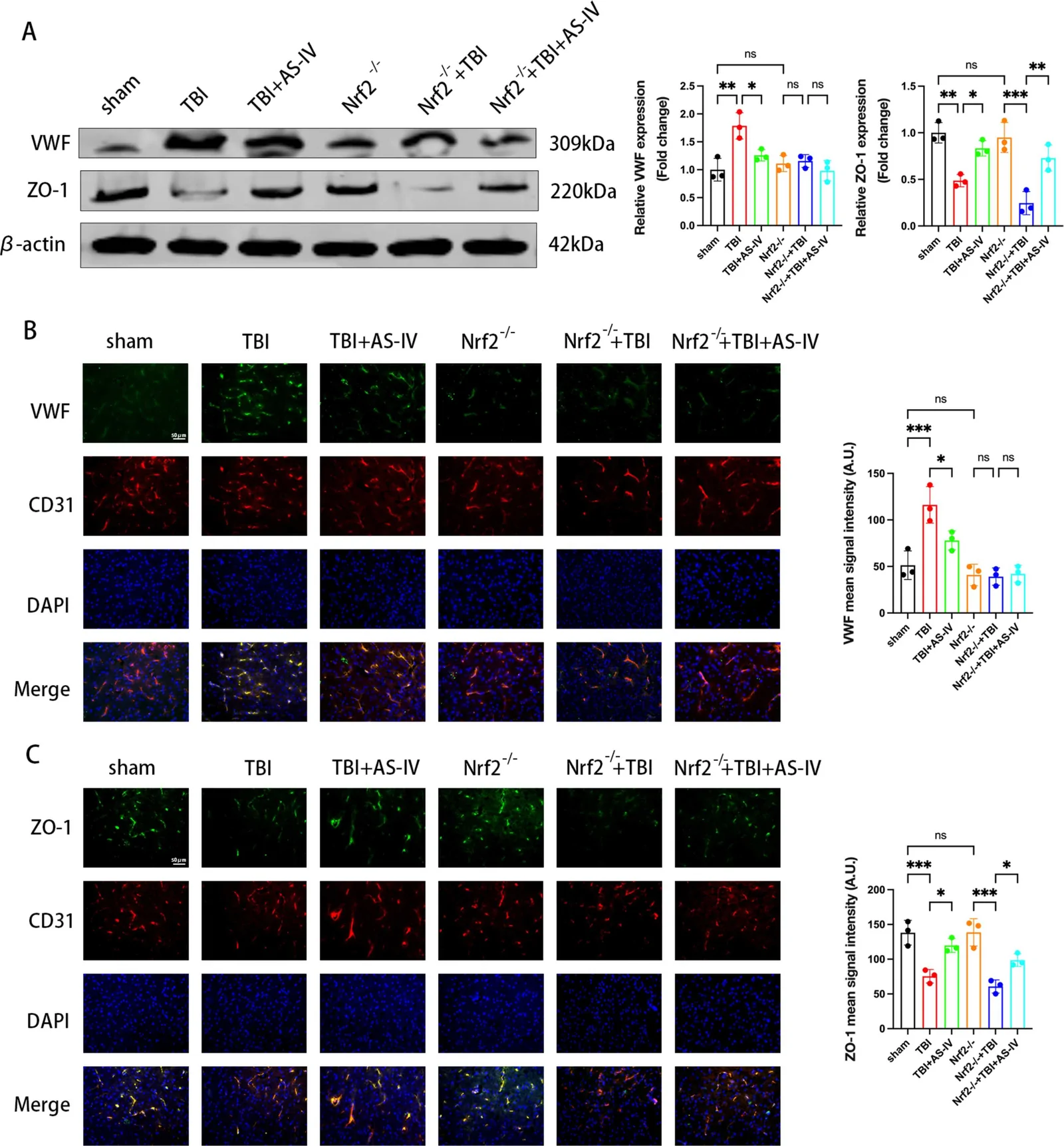
Nrf2 deficiency abrogates AS-IV mediated regulation of VWF expression after TBI. (A) Western blot and quantitative analysis of VWF and ZO−1 protein levels in the pericontusional cortex (n = 3 per group). (B–C) Double immunofluorescence staining of VWF or ZO−1 (green) with CD31 (red) in cortical sections, with quantitative analysis of fluorescence intensity within CD31 positive vascular structures (n = 3 per group). Scale bar, 50 μm. Data are presented as mean ± SD. *p < 0.05, **p < 0.01, ***p < 0.001; ns, not significant.

VWF and ZO−1 expression in the pericontusional cortex were examined in WT and Nrf2⁻/⁻ mice after TBI. Western blot analysis showed that in WT mice, TBI markedly increased VWF protein expression and reduced ZO−1 levels in the pericontusional cortex. AS-IV treatment significantly attenuated VWF upregulation and restored ZO−1 expression. In Nrf2⁻/⁻ mice, TBI still reduced ZO−1 expression, whereas VWF levels were not markedly increased after injury. In Nrf2⁻/⁻ mice, AS-IV restored ZO−1 expression but failed to modulate VWF protein levels (Fig. 5A). Immunofluorescence analysis further examined VWF and ZO−1 expression within CD31-positive vascular structures in the pericontusional cortex. In WT mice, TBI increased vascular VWF immunoreactivity and decreased ZO−1 fluorescence intensity, both of which were reversed by AS-IV treatment. In Nrf2⁻/⁻ mice, AS-IV restored ZO−1 expression after TBI; however, vascular VWF expression was neither markedly induced by TBI nor responsive to AS-IV administration (Fig. 5B–C).

## Discussion

The present study systematically evaluated the protective effects of AS-IV after TBI and demonstrated that its vascular protective effects rely on Nrf2 signaling. Further analyses suggest that VWF may participate in this Nrf2-dependent vascular protective mechanism. These findings reveal a potential mechanism underlying the protective effects of AS-IV after TBI and provide new insights into the role of Nrf2 signaling in TBI.

AS-IV exerts neuroprotective effects in several neurological disorders, whereas evidence in the context of TBI remains relatively limited. In addition, accumulating studies suggest that AS-IV can activate the Nrf2 signaling pathway and exert protective effects through Nrf2-related mechanisms in multiple disease models (Yao et al., 2023). However, whether the neuroprotective effects of AS-IV in TBI involve Nrf2 signaling has not been systematically investigated. In the present study, behavioral and histological analyses demonstrated that AS-IV significantly improved neurological function and attenuated neuronal injury following TBI. Concurrently, AS-IV promoted Nrf2 nuclear translocation and increased the expression of downstream proteins. Collectively, these findings indicate that activation of the Nrf2 signaling pathway contributes to the neuroprotective effects of AS-IV following TBI.

AS-IV exhibits a broad range of pharmacological activities, including anti-inflammatory, antioxidant, anti-fibrotic, and anti-tumor effects (Liang et al., 2023). Several studies have reported that AS-IV exerts protective effects on the vasculature, for example by improving vascular endothelial dysfunction through suppression of inflammatory signaling pathways and reduction of oxidative stress (Leng et al., 2018, Zhang et al., 2021). However, studies investigating the molecular mechanisms by which AS-IV exerts protective effects in TBI remain limited, and the few available reports have mainly focused on its anti-inflammatory effects (Yang et al., 2014, Zhao et al., 2024). We performed a more comprehensive evaluation of the effects of AS-IV after TBI. Our results demonstrate that AS-IV not only attenuates inflammation and oxidative stress, but also reduces brain edema, preserves blood-brain barrier integrity, and improves cerebral blood flow in experimental TBI.

Given the central role of Nrf2 signaling in TBI pathogenesis (Hong et al., 2010), we utilized Nrf2-deficient mice to systematically evaluate its contribution to AS-IV-mediated neuroprotection. Notably, the anti-inflammatory and antioxidant actions of AS-IV were not completely abolished in Nrf2-deficient mice, indicating that these protective effects are associated with Nrf2 signaling but are not strictly dependent on it. This observation likely reflects the pleiotropic pharmacological properties of AS-IV. Previous studies have shown that AS-IV can modulate multiple signaling pathways, including NF-κB (Zhang and Frei, 2015), PI3K-Akt (Wang et al., 2024), and MAPK (Hsieh et al., 2022), all of which are known to participate in the regulation of inflammatory responses and oxidative stress. This suggests that AS-IV may exert its effects through a broader signaling network rather than a single pathway.

Vascular dysfunction represents a major component of secondary injury after TBI (Whitehead et al., 2024). In the present study, the vascular protective effects of AS-IV were almost completely abolished in Nrf2-deficient mice, suggesting that this protective action is highly dependent on Nrf2 signaling. This finding appears difficult to reconcile with previous reports. AS-IV has been reported to improve vascular endothelial function through activation of the PI3K/Akt signaling pathway (Shi et al., 2023). Transcriptomic analysis was performed to compare gene expression between WT and Nrf2-deficient mice after TBI, which revealed von Willebrand factor (VWF) as a vascular-related gene markedly altered in Nrf2-deficient mice. VWF is a multimeric glycoprotein synthesized and stored in endothelial cells and released upon endothelial activation, where it mediates platelet adhesion during vascular injury. Elevated VWF levels are widely considered a marker of endothelial dysfunction (Lopes da Silva and Cutler, 2016, Nightingale and Cutler, 2013). In the present study, VWF expression increased after TBI in WT mice but not in Nrf2-deficient mice, and AS-IV reduced the TBI-induced increase in VWF only in WT mice. These findings suggest that disruption of VWF regulation in Nrf2-deficient mice may contribute to the loss of vascular protection by AS-IV after TBI.

This study has several limitations. First, although VWF expression was associated with Nrf2-dependent vascular protection after TBI, the present study did not establish a direct regulatory or functional relationship among AS-IV, Nrf2, and VWF. Further clarification of this relationship would strengthen the mechanistic understanding of AS-IV-mediated vascular protection after TBI. Second, functional assessments were limited to the acute phase up to 3 days after TBI, and whether the beneficial effects of AS-IV persist into the chronic phase remains unclear.

## Conclusion

In conclusion, AS-IV exerts neuroprotective effects after TBI, and its vascular protective actions rely on Nrf2 signaling. VWF may contribute to this Nrf2-dependent process.

## CRediT authorship contribution statement

**Longlong Tian:** Validation. **Guozhu Sun:** Supervision, Resources, Project administration, Funding acquisition, Conceptualization. **Boyu Sun:** Data curation. **Mingkang Li:** Writing – review & editing. **Shiyao Feng:** Validation, Methodology, Investigation.

## Funding

This research was supported by the Hebei Provincial Natural Science Foundation of China (Grant No. H2024206437).

## Declaration of Competing Interest

The authors declare no competing interests.
